# Testing for SES differences in the responsiveness of educational expectations in a twin design

**DOI:** 10.1371/journal.pone.0290454

**Published:** 2023-08-24

**Authors:** Mirko Ruks

**Affiliations:** Faculty of Sociology, Bielefeld University, Bielefeld, Germany; The Ohio State University, UNITED STATES

## Abstract

In this article I test whether students’ educational expectations respond to prior academic performance and whether this responsiveness varies by socio-economic status (SES). The responsiveness of high-SES students’ expectations may be lower as suggested by the compensatory advantage mechanism or higher because of alienation processes of low-SES students. However, the association between achievement and expectations may be in part spurious because of unobserved social and genetic confounders. This issue is largely ignored by previous research. Therefore, in this paper I estimate behavioral genetic twin models that take into account the possible confounding of the responsiveness of expectations to performance by unobserved genetic and social influences. While students’ expectations respond to prior performance, this responsiveness is reduced by more than half once unobserved genetic and social confounders are accounted for. Also, SES differences in responsiveness to performance are completely accounted for by high-SES students’ expectations being less responsive to prior levels of cognitive ability. So, this study shows the relevance of taking different types of confounding into account when studying the formation of educational expectations.

## Introduction

Inequality of educational opportunity (IEO), defined as “differences in level of educational attainment according to social background” [1] is a well documented phenomenon in social inequality research [2]. However, it’s mechanisms are still not fully understood. Previous research conceptualizes educational expectations as the “strategic center” of the educational attainment process [3]. Indeed, despite of rising educational expectations over time [4], students’ expectations are a strong predictor of educational success [5–7]. Given this relevance of educational expectations for educational attainment, it is important to understand how expectations are formed. One part of the research asks how students’ expectations respond to signals of academic potential [8–10] and whether this responsiveness varies by socio-economic status [11, 12]. On the one hand, high-SES students may be less responsive as a high SES can compensate for prior bad performance [13]. On the other hand, high-SES students may be more responsive as low-SES students may anticipate their possible educational failure or feel culturally alienated from educational institutions, decoupling their expectations from any signal of academic potential [12].

Following this line of research, in this study I investigate whether students’ expectations respond to prior academic performance and whether this responsiveness varies by SES. However, the relationship between academic performance and expectations may be partly biased by unobserved social or genetic factors. On the one hand, there are social factors at the family level, such as family SES, school composition or neighborhood context, that have an effect on students’ expectations [3, 14, 15] and are also related to academic performance [16–18], thus possibly confounding the observed association. On the other hand, there is robust evidence of genetic influences on academic performance [19] and there are also genetic influences on educational expectations [20] which can be explained by an adjustment of expectations based on genetically influenced traits. As genetic dispositions related to cognitive and educational outcomes also have an effect on educational expectations [21], there is a risk of genetic confounding of the association of expectations and performance. So, in light of the possible confounding due to unobserved social and genetic factors, it is crucial to control for these confounders when studying the formation of expectations. While there are some studies using panel data to apply more robust methods for causal inference [12, 22, 23], so far no study of the formation of educational expectations has explicitly controlled for social and genetic confounding.

The main contribution of this study is to address this issue by controlling not only for unobserved social and genetic confounders but also for cognitive ability as one important observed confounder. Using panel data (N = 1033 twin pairs, age 10–14) from the German TwinLife study [24], I estimate biometric ACE-beta twin models [25] that control for social and genetic confounding by estimating these confounders as latent variables within the model, providing an direct estimate of social and genetic confounding. To control for these types of confounding is crucial as they may not only affect the estimates of the responsiveness of educational expectations to academic performance but also of the SES differences of this responsiveness.

## Theoretical background

Educational expectations can be distinguished from educational aspirations. Both are “prefigurative orientations composed of specific beliefs about one’s future trajectory” [26]. Aspirations are conceptualized as unconstrained idealistic goals [26] referring to “the level of education that an individual would ideally like to obtain” [27], while expectations refer to more realistic beliefs that take into account the subjective probability of success [27]. Different to aspirations, expectations are more responsive to indicators of academic potential in particular and the educational opportunity structure in general [27] and are more predictive for educational outcomes [28, 29]. In the remainder of this section I will discuss theoretical arguments about the responsiveness of educational expectations and why the responsiveness may vary by SES.

### The responsiveness of educational expectations

According to the Wisconsin model of status attainment [30, 31], students’ educational expectations are the “strategic center” of educational attainment and primarily shaped by SES and the expectations held by significant others, such as parents or teachers. This adoption of expectations held by significant others is considered to be the main mechanism of expectation formation [31] so that educational expectations are assumed to be a stable mental construct that is formed early in life and hardly changes over the life course [32].

Contrary to this perspective, rational choice approaches [32–35] propose a more dynamic perspective on the formation of educational expectations. Here, the educational attainment process is conceptualized as a sequence of educational decisions [36] where educational choices and expectations as “anticipated choices” [37] are formed in a process of rational cost-benefit calculation taking into account the (subjective) utility and the expected probability of the different educational outcomes. The expected probabilities depend on the perceived opportunity structure in general and the students’ perceived academic potential in particular [32–34].

During their educational career, students accumulate information about their academic potential which allows them to constantly re-evaluate their expected success probabilities which may result in an adjustment of their educational expectations [32–35, 38]. Signals of academic potential come from a “variety of sources” like school, peers or family [38]. However, institutionalized indicators of academic performance like school grades belong to the most relevant signals of academic potential [12, 39]. They are relevant for future success within the educational system as they are closely linked to the educational reward structure, they are directly observable and provide a benchmark to assess the own relative position via the comparison to other students [40–43].

Previous research has tested whether students’ expectations are updated based on different signals of academic potential. [9, 12] shows for the US that students adjust their expectations based on track placement and academic performance. Also for the US, [8, 10] report that students adjust their expectations based on prior academic performance while [39] and [44] find only a modest responsiveness of students’ expectations. For Germany, [45] and [23] show that students adjust their expectations downwards after attending a lower school track. However, [22] show that the responsiveness is mainly driven by unobserved confounding. For Spain, [46] shows for Spain that being sorted into a low-ability group in school is followed by a downwards adjustment of students’ expectations. So, the following hypothesis can be stated:

*H*_1_: *Students’ educational expectations respond to prior academic performance*.

### SES differences in the responsiveness of educational expectations

How and why may the responsiveness of students’ educational expectations to academic performance differ by SES? According to the compensatory advantage (CA) mechanism “life course trajectories of individuals from privileged backgrounds are less dependent on prior negative outcomes” [13]. For the formation of educational expectations, the “prior negative outcome” can be conceptualized as a prior bad academic performance. So, high-SES students’ expectations are expected to be less responsive to prior bad performance than low-SES students’ expectations.

This can be explained by the theory of relative risk aversion (RRA) according to which educational decision making is guided by the motive to avoid social downward mobility [33]. To achieve this goal, high-SES students need to attain higher levels of education than low-SES students which is reflected in higher educational expectations, irrespective of their academic performance. As low-SES students do not need to attain high levels of education, they are more likely to adjust their expectations downwards in response to bad performance. So, the SES gap in expectations should be strongest among students with prior bad performance since here the risk of social downward mobility is greatest [11]. High-SES students do not only have the incentive to ignore negative signals of academic potential, their families also have the necessary resources to successfully realize their high expectations despite these negative signals [13]. For example, high-SES parents can guide their offspring better through the educational institutions, provide better learning materials, pay private tutoring or pressure educational gatekeepers [18, 47].

A contrasting perspective according to which low-SES students’ expectations are less responsive can be derived from the concept of self-elimination [48]. Self-elimination is one mechanism of social exclusion and refers to the situation where individuals adjust their expectations to their perceived chances of success as they realize the constraints associated with their socio-economic position or because they do not feel at ease in specific social settings where they are not familiar with specific cultural norms [48]. So, a socio-economic and a cultural explanation of self-elimination can be distinguished. Following the socio-economic explanation, low-SES families lack the necessary resources to support their offspring so that low-SES students are less likely to attain high levels of education even when they are well performing [12]. Within an “unconscious estimation of their objective probabilities of success” [49], low-SES students may anticipate their possible educational failure. As a consequence their expectations may become less responsive to signals of academic potential [12, 50]. Following the cultural explanation, the cultural codes of the educational system resemble the cultural norms and values of middle- and high-classes [51]. While the “[l]anguage styles and cultural practices of the socioeconomically advantaged are rewarded” [2], low-SES students are not familiar with the cultural codes of the educational system and may feel like guests in the educational institutions. This may result in an alienation between low-SES students and educational institutions in general [52] and a lower responsiveness of low-SES students’ expectations in particular [12, 50].

Some studies tested for SES differences in the responsiveness of educational expectations. [45] show for Germany that the downward adjustment of students’ expectations after changing to the nonacademic track is less pronounced for high-SES students. A similar result is reported by [12] for the US and in an international comparison for 11 OECD countries [11] show that SES differences are greatest among low performing students. Based on the discussion in this section, the following hypotheses can be formulated:

*H*_2_: *The responsiveness of educational expectations to prior performance is lower for high-SES students than for low-SES students*.
*H*_3_: *The responsiveness of educational expectations to prior performance is higher for high-SES students than for low-SES students*.

## Data

The study uses data from the German TwinLife survey [24] which is a longitudinal survey of four birth cohorts of same-sex twins and their biological families that started in 2014. TwinLife applies a register-based probability sampling design and includes families along the whole range of the socio-economic spectrum in Germany [53]. The TwinLife survey received an ethics approval from the German Psychological Association (protocol number: RR 11.2009 and RR 09.2013), complying with the ethical standards of the 1964 Helsinki declaration and its later amendments. Informed oral consent was obtained and recorded as part of the study procedure during the household interviews. For minors at least one parent or guardian had to give consent. Participant’s consent was recorded by the interviewer in the interview protocol which was in line with the German law and was reported to the ethics committee. An identification of the participants by the researchers is not possible. For the analysis I focus on the second birth cohort (born 2003/04) using data from the first two survey waves conducted in 2014–16 (age range: 10–12) and 2016–18 (age range: 12–14), respectively.

Students’ educational expectations are measured in the second wave. It is operationalized by weighting the desired school-leaving qualification (1: lower (“Hauptschulabschluss”, after 9/10 years of schooling); 2: intermediate (“Realschulabschluss”, after 10 years of schooling), 3: higher (“Abitur”, after 12/13 years of schooling). While the first two provide access to a vocational training, the latter additionally allows to study at university. with the perceived probability of success for the desired degree (0–100%). By weighting the educational aspirations with the perceived probability of success, the operationalization closely follows the theoretical distinction between idealistic aspirations and realistic expectations [26, 54]. The variable is standardized to zero mean and unit variance.

Academic performance is measured in the first wave by the average grade of the subjects Mathematics and German. Both are core subjects in the German school curriculum so the performance in both subjects is central for future educational success. Taking the average of both subjects is important as it represents the overall performance in the core subjects. The grades are reverse coded so that a higher average grade indicates better performance. To account for different school types, I residualize the average grades for school type (see the first section in S1 Appendix for the coding scheme of the school type variable). However, a robustness check shows that results do not change when using school grades that are not residualized for school type (see Table A6 in S1 Appendix).

Parental SES is measured by parental education as there is robust evidence that parental education is the SES dimension that has the strongest effect on children’s educational and cognitive outcomes [47]. Following previous research using the same data [55, 56], parental education is measured by years of education using the coding scheme by the german socio-economic panel (SOEP). For parents with different years of education, the mean value is used. For a better interpretation of the moderation results, the variable is standardized with mean zero and unit variance. A robustness check shows that results do not change when using a composite score for SES including parental education, occupational status and income (see Table A5 in S1 Appendix).

As previous studies have shown, cognitive ability has a positive effect on educational expectations [57] and is also strongly correlated with academic performance [58]. Therefore, it is necessary to control for cognitive ability when estimating the responsiveness of expectations to performance. Cognitive ability is measured in the first wave by a computer administrated version of the widely applied Culture Fair Test [59] that measures fluid intelligence. Compared to crystalized intelligence (i.e. learned knowledge and skills), fluid intelligence measures the ability to acquire skills and knowledge. Based on the sum scores of all four subdimensions of the CFT—figural reasoning (15 items), figural classification (15 items), matrices (15 items) and reasoning (11 items)—provided in the TwinLife data an indicator of cognitive ability is constructed by estimating a CFA.

To avoid overestimating the shared environmental effects [60], I residualize the variables for sex and age from the first wave. The Full Information Maximum Likelihood Estimator [61] is used to deal with missing data. So, an observation is used for the analysis if it has at least one non-missing value on the analysis variables. For the moderator parental education missing data need to be listwise deleted, which is only the case for 12 observations. The final sample size is N = 1029 twin pairs. A descriptive overview of the final sample is provided in Table 1.

**Table 1:** Summary statistics based on long-formatted data (one row = one twin).

Variable	N	Mean	SD	Min	Max
Avg. Grades	1692	0	0.76	-3.05	1.82
Expectations	1378	0	1	-3.87	1.42
Parental Education	2058	0	1	-2.19	2.07
Sex (Ref.: Male)	2058	0.52	0.5	0	1
Age	2058	11	0.32	10	12
IQ	2032	0	1	-3.56	2.7
N (Twins)	2058				
N (Twin Pairs)	1029				

## Methods

The statistical analysis consists of several biometric twin models [62, 63]. By comparing genetically identical monozygotic (MZ) and dizygotic (DZ) twins that share on average 50% of their genes, the variance of a variable can be decomposed into effects of four components: 1) Additive genetic influences, i.e. the effects of different alleles simply “add up” (A), 2) Non-additive genetic influences, i.e. interactions between alleles at the same locus (dominance) or across different loci (epistasis) (D), 3) Environmental influences shared by the twins, such as family SES or neighborhood environment (C), 4) Environmental influences not shared by the twins, such as peer effects, including measurement error (E). As a model with all four components is not identified [63], model selection is is usually guided by inspecting the twin correlations. If the DZ twin correlations are smaller than half the MZ twin correlations, an ADE model is appropriate, otherwise the ACE model [62]. As shown in Table A1 in S1 Appendix, the DZ twin correlations are not smaller than half the MZ twin correlations, so the ACE model is appropriate. The twin models are estimated as structural equation models with the ACE components as latent factors with a variance fixed to 1 and the path coefficients as freely estimated parameters [63]. The identification of the estimates of the twin models rests on the following assumptions [64]. 1) The equal environment assumption (EEA) states that the environmentally caused similarity is equal for MZ and DZ twins. A violation of the EEA would lead to an overestimation of the genetic effects and an underestimation of the shared environmental effects. However, the validity of this assumption has been confirmed for various traits [62] and even a violation does not affect the results substantially [65]. 2) There is no assortative mating for the studied phenotypes. Espectially for educational and cognitive outcomes this assumption is likely violated [66], resulting in an underestimation of the genetic effects [64]. However, a robustness check shows that the main results do not change when accounting for varying degrees of assortative mating (see Table A4 in S1 Appendix). 3) There are no gene-environment interactions (G×E) or correlations (rGE). As I estimate moderation models, I explicitly test for the presence of G×E and per design I control for rGE between the performance and expectations and by controlling for the main effect of the moderator, I also control for rGE between SES and both variables in the model. Only results of the best fitting models are reported. The model comparisons are summarized in Tables A2 and A3 in S1 Appendix. To estimate the models, I use the R package twinflex [67] which is a wrapper function for the the OpenMx in R [68].

**Fig 1:**
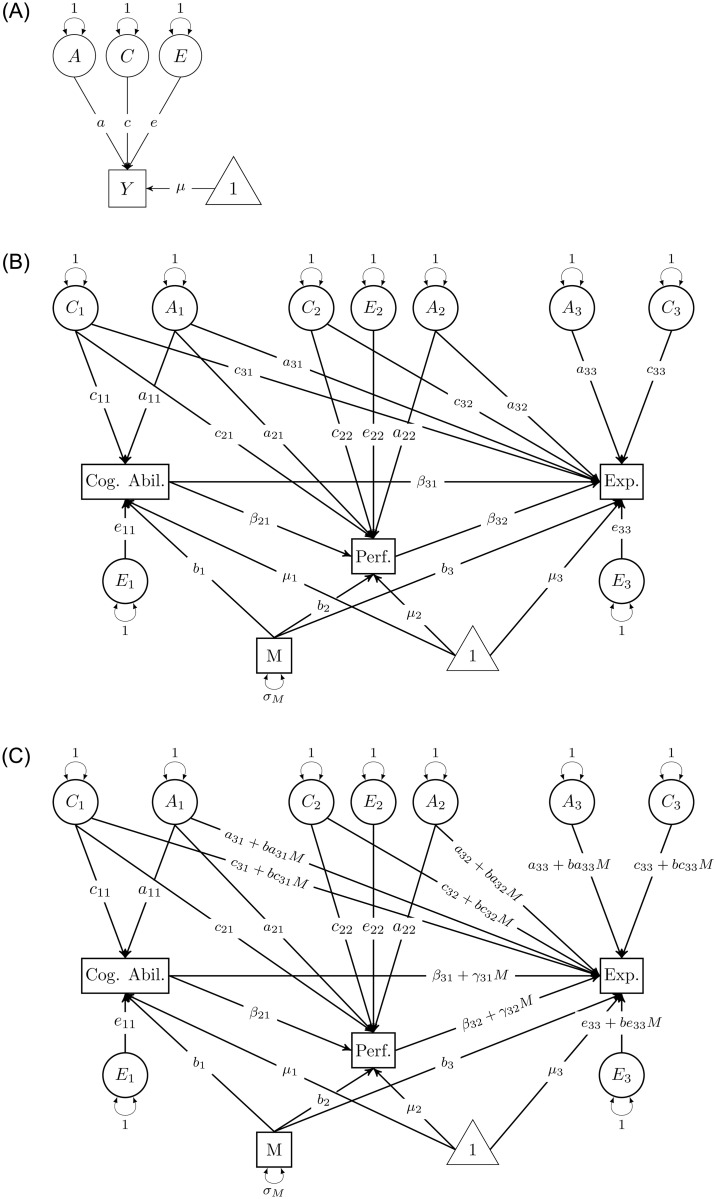
Path diagrams of estimated twin models. Note: M = Parental Education. A: Univariate model. B: Multivariate model. C: Moderation model.

The statistical analysis consists of three steps: In the first step (see panel A in Fig 1), univariate twin models are estimated for the average grades (M1a), educational expectations (M1b) and cognitive ability (M1c). Here, the variance of the three outcomes is decomposed into effects of the ACE components providing a first overview of the relevance of genetic and environmental influences. However, it remains open whether the variables are influenced by the same genetic and shared environmental influences which would introduce bias to the observed association or whether the genetic and shared environmental factors are independent from each other.

Therefore, in the second analysis step, multivariate ACE-beta models [25] are estimated to test whether expectations respond to prior academic performance while controlling for confounding due to unobserved genetic and shared environmental influences and cognitive ability. The model is shown in panel B in Fig 1. The parameters *a*_22_, *c*_22_ and *e*_22_ refer to the ACE effects on performance. The parameters *a*_32_ and *c*_32_ denote to what extend genetic and shared environmental factors associated with academic performance also influence expectations and the parameter *β*_32_ refers to the responsiveness of educational expectations to prior performance. Equivalently for cognitive ability, the parameters *a*_11_, *c*_11_ and *e*_11_ describe the genetic and environmental influences on cognitive ability and the parameters *a*_21_, *c*_21_ and *a*_31_, *c*_31_ show whether these genetic and shared environmental factors also affect performance or expectations, respectively. The parameters *β*_21_ and *β*_31_ refer to the phenotypic effect of cognitive ability on performance and expectations, respectively. Finally, the residual variance of expectations is decomposed into genetic and environmental influences (*a*_33_, *c*_33_, *e*_33_). There are SES main effects on cognitive ability (*b*_1_), performance (*b*_2_) and expectations (*b*_3_). The intercepts are depicted by the pathways coming from the triangle. So, the parameter of interest is *β*_32_, as it is the estimate of the responsiveness of expectations to prior performance while accounting for possible confounding by unobserved genetic and shared environmental factors and cognitive ability.

Conceptually, the ACE-beta model is comparable to the fixed-effects model with MZ twins (MZ-FE) [25]. However, it comes with the advantage that it explicitly models the unobserved genetic and shared environmental confounders, thus informing about the relative contribution of unobserved genetic and shared environmental confounders and the actual effect to the association of performance and expectations. As the ACE-beta model accounts for unobserved genetic and shared environmental confounders, like the MZ-FE model it only needs to assume exogeneity with respect to the non-shared environmental influences [25]. Therefore, I control for cognitive ability which is a major factor varying within a twin pair and could possibly bias the estimated responsiveness to performance.

In total, the second analysis step consists of four models (M2a-M2d) with different assumptions about the confounding of the association. Comparing the model results provides insights into the social and genetic confounding of the responsiveness of educational expectations to performance. Model M2a assumes no confounding at all and estimates only the responsiveness of educational expectations to performance (*β*_32_). Model M2b additionally controls for confounding by genetic and shared environmental factors associated with performance, showing how much of the association between expectations and achievement is accounted for by social and genetic confounding. Model M2c controls also for cognitive ability and genetic and shared environmental factors associated with cognitive ability, showing whether cognitive ability confounds the estimated responsiveness and how much of the unobserved confounding is explained by social and genetic factors associated with cognitive ability. Finally, model M2d controls for SES, showing how much of the shared environmental confounding is accounted for by SES.

The third analysis step consists of the moderation analysis to test for SES differences in the responsiveness of educational expectations to performance (see panel C in Fig 1). Here, *β*_32_ + *γ*_32_*M* is the pathway of interest and refers to the moderation of the responsiveness of educational expectations to academic performance by SES. *β*_32_ is the main effect and denotes the responsiveness of expectations to academic performance when *M* = 0, i.e. for an average level of SES. *γ*_32_ is the interaction effect and indicates whether and, if so, how strong the responsiveness of educational expectations to academic performance varies by SES. The same logic applies to the other moderated effects. As the estimated SES differences in the responsiveness to performance may be confounded by unmodelled SES differences in the effects of other predictors, all incoming pathways for educational expectations are allowed to vary by SES. Two moderation models are estimated. In model M3a, only the pathways coming from genetic and shared environmental factors associated with performance, the responsiveness to performance and the effects of the ACE factors unique to expectations are moderated. In model M3b, also the effects of genetic and shared environmental factors associated with cognitive ability and the responsiveness to cognitive ability are allowed to vary by SES. Note that the main results do not change when controlling for prio educational expectations (see Table A7 in S1 Appendix).

## Results

### Univariate analysis

The results of the univariate twin models for academic performance (M1a) and educational expectations (M1b) and cognitive ability (M1c) are shown in Table 2 and provide a first overview of the relevance of genetic and environmental influences for the three variables. The standardized variance components can be calculated following path tracing rules. For example, the standardized genetic variance (“heritability”) is calculated as $a_{11}^{2}/\left(a_{11}^{2}+c_{11}^{2}+e_{11}^{2}\right)$. There are genetic and environmental influences on all three variables. For performance (M1a), genetic influences are strongest, as the proportion of variance in performance accounted for by genetic differences, i.e. the heritability, is 54%, while influences of shared and non-shared environmental factors are somewhat weaker, explaining about 23%, respectively. For educational expectations (M1b), genetic influences are clearly strongest, accounting for 67% of the variance, indicating that students adjust their expectations based on genetically influenced traits [21]. Non-shared environmental factors explain 26% and the effect of shared environmental factors—significant only at *α* = 10%—accounts for the remaining 7% of the variance. Finally, for cognitive ability (M1c), genetic and non-shared environmental influences are comparable, as the former explain 40% of the variance and the latter 37%. Shared environmental effects are little weaker, explaining 23% of the variance. While the univariate models clearly show the presence of genetic and shared environmental influences, it remains open to what degree the three variables are influenced by the *same* genetic and shared environmental factors which could introduce bias to the estimated responsiveness of expectations. This question is addressed in the second analysis step.

**Table 2:** Results of best-fitting univariate models. Standard errors in parentheses.

	M1a	M1b	M1c
*a*_11_	0.56*** (0.04)	0.82*** (0.06)	0.63*** (0.06)
*c*_11_	0.37*** (0.06)	0.27^+^ (0.15)	0.48*** (0.07)
*e*_11_	0.37*** (0.01)	0.51*** (0.02)	0.6*** (0.02)
-2LL	3484.65	3616.54	5423.69
N	889	717	1024

*p* < 0.001:***; *p* < 0.01:**; *p* < 0.05:*; *p* < 0.10:^+^.

### Multivariate analysis

The results of the multivariate ACE-beta models M2a-M2d calculated in the second analysis step are presented in Table 3. In M2a, the responsiveness of expectations to prior academic performance is estimated without controlling for any genetic and shared environmental confounding, cognitive ability and SES. There is a strong and significant effect of prior academic performance on expectations (*β*_32_ = 0.46, *p* < 0.001) suggesting expectations being highly responsive to prior performance. The genetic and environmental influences on cognitive ability and performance are comparable to the univariate results. For expectations, *c*_33_ can be fixed to zero, meaning that there are no unique shared environmental influences and all shared environmental influences are mediated by prior performance.

**Table 3:** Results of best-fitting multivariate models.

	M2a		M2b		M2c		M2d	
Exp.								
*a*_33_	0.76***	(0.03)	0.75***	(0.03)	0.67***	(0.04)	0.65***	(0.04)
*c*_33_	0		0		0		0	
*e*_33_	0.51***	(0.02)	0.5***	(0.02)	0.5***	(0.02)	0.5***	(0.02)
Avg. Grades → Exp.								
*a*_32_	-		0.24*	(0.11)	0		0	
*c*_32_	-		0.23*	(0.1)	0.19*	(0.09)	0	
*β*_32_	0.46***	(0.04)	0.16^+^	(0.09)	0.16*	(0.07)	0.21***	(0.06)
Cog. Abil. → Exp.								
*a*_31_	-		-		0.33**	(0.11)	0.31***	(0.09)
*c*_31_	-		-		0.17^+^	(0.1)	0	
*β*_31_	-		-		0.09^+^	(0.05)	0.08^+^	(0.05)
Avg. Grades								
*a*_22_	0.56***	(0.04)	0.57***	(0.04)	0.48***	(0.05)	0.48***	(0.04)
*c*_22_	0.37***	(0.06)	0.37***	(0.06)	0.33***	(0.06)	0.28***	(0.06)
*e*_22_	0.37***	(0.01)	0.37***	(0.01)	0.37***	(0.01)	0.37***	(0.01)
Cog. Abil. → Avg. Grades								
*a*_21_	-		-		0.23**	(0.08)	0.23***	(0.06)
*c*_21_	-		-		0.12^+^	(0.07)	0	
*β*_21_	-		-		0.11***	(0.03)	0.11***	(0.03)
Cog. Abil.								
*a*_11_	0.63***	(0.06)	0.63***	(0.06)	0.63***	(0.06)	0.62***	(0.06)
*c*_11_	0.48***	(0.07)	0.48***	(0.07)	0.47***	(0.07)	0.38***	(0.08)
*e*_11_	0.6***	(0.02)	0.6***	(0.02)	0.61***	(0.02)	0.61***	(0.02)
Par. Educ.								
*b*_1_	-		-		-		0.32***	(0.03)
*b*_2_	-		-		-		0.2***	(0.02)
*b*_3_	-		-		-		0.3***	(0.04)
-2LL	15311.13		15292.67		14924.93		14684.51	
N	1029		1029		1029		1029	

Standard errors in parentheses. Parameters with ‘0’ without standard error could be fixed to 0. Significance: *p* < 0.001:***; *p* < 0.01:**; *p* < 0.05:*; *p* < 0.10:^+^.

In model M2b, genetic and shared environmental factors associated with academic performance are allowed to influence expectations as well. There is a strong and significant effect of genetic (*a*_32_ = 0.24, *p* < 0.05) and shared environmental factors (*c*_32_ = 0.23, *p* < 0.05) associated with performance on expectations. So, these unobserved genetic and shared environmental factors influencing both, performance and expectations, confound the responsiveness to performance estimated in M2a. As a result, the estimated responsiveness to performance drops to *β*_32_ = 0.16 and is only significant at *α* = 10%. This means, that based on model M2b a substantial part of around about 65% of the responsiveness to performance estimated in M2a can be attributed to genetic and shared environmental confounding.

Model M2c, additionally controls for the phenotypic effect of cognitive ability and the effects of unobserved genetic and shared environmental factors associated with cognitive ability. As shown in Table 3, the genetic factor associated with cognitive ability has a strong and significant effect on performance (*a*_21_ = 0.23, *p* < 0.01) and expectations (*a*_31_ = 0.33, *p* < 0.01). After controlling for these genetic effects, the effect of genes associated with performance and independent of cognitive ability on expectations (*a*_32_) can be fixed to zero, meaning that the genetic confounding of the responsiveness of expectations to performance is fully explained by a common genetic factor for cognitive ability and performance. Shared environmental factors associated with cognitive ability also affect performance (*c*_21_ = 0.12, *p* < 0.10) and expectations (*c*_31_ = 0.17, *p* < 0.10), leading to a small reduction of the effect of shared environmental factors associated with performance on expectations to *c*_32_ = 0.19 (*p* < 0.05). Finally, controlling for unobserved genetic and shared environmental factors, cognitive ability itself has a positive effect on performance (*β*_21_ = 0.11, *p* < 0.001) and expectations (*β*_31_ = 0.09, *p* < 0.10). Controlling for cognitive ability, however, does not affect the estimated responsiveness to performance, suggesting independent effects of cognitive ability and performance on expectations. But controlling for cognitive ability increases the precision of the estimate, as the responsiveness to performance is now significant at *α* = 0.05.

The final model M2d additionally controls for SES. This leads to a substantial drop in the estimated effects of the unobserved shared environmental factors. The effect of unobserved shared environmental factors on performance (*c*_22_ = 0.28, *p* < 0.001) and cognitive ability (*c*_11_ = 0.38, *p* < 0.001) is substantially reduced. SES completely accounts for the effect of shared environmental factors associated with performance on expectations (*c*_32_) and the effects of shared environmental factors associated with cognitive ability on performance (*c*_21_) and expectations (*c*_31_), as these three parameters can be fixed to zero. Therefore, all shared environmental confounding of the responsiveness to performance is accounted for by SES. So, based on the final multivariate ACE-beta model M2d, students adjust their expectations based on prior performance (*β*_32_ = 0.21, *p* < 0.001). This result is in line with hypothesis *H*_1_. However, the responsiveness to prior performance is substantially confounded by unobserved genetic and shared environmental factors as about 54% of the estimated responsiveness in M2a (*β*_32_ = 0.46, *p* < 0.001) is explained by confounding.

### Moderation analysis

**Fig 2:**
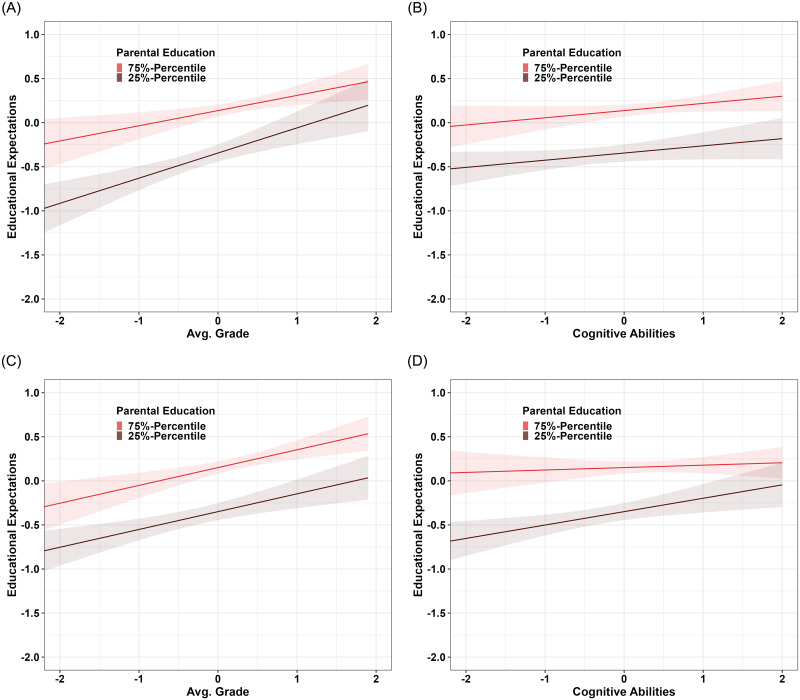
Prediction plots of moderation analysis. A: Responsiveness to Avg. Grades based on model M3a. B: Responsiveness to cognitive ability based on model M3a. C: Responsiveness to Avg. Grades based on model M3b. D: Responsiveness to cognitive ability based on model M3b.

In the third analysis step I estimate moderated ACE-beta models to assess whether the responsiveness of students’ expectations to prior performance varies by SES. The model results are summarized in Table 4 and a visualization of the interactions is shown in Fig 2. In model M3a, the effects of the ACE components unique to expectations, the genetic and shared environmental factors associated with performance and the responsiveness to performance are allowed to vary by SES. In the best-fitting model, only two paths vary by SES. The effect of genes unique to expectations is negatively moderated by SES. While for students with an average SES level, the effect is *a*_33_ = 0.66 (*p* < 0.001), a 1 SD increase in SES leads to a reduction of the effect by 0.08 points (*ba*_33_ = −0.08, *p* < 0.001).

**Table 4:** Results of best-fitting moderation models.

	M3a		M3b	
Exp.				
*Main Effects*				
*a*_33_	0.66***	(0.04)	0.66***	(0.04)
*c*_33_	0		0	
*e*_33_	0.5***	(0.02)	0.5***	(0.02)
*Interaction Effects*				
*ba*_33_	-0.08**	(0.03)	-0.09***	(0.03)
*bc*_33_	0		0	
*be*_33_	0		0	
Avg. Grades → Exp.				
*Main Effects*				
*a*_32_	0		0	
*c*_32_	0		0	
*β*_32_	0.22***	(0.06)	0.2***	(0.05)
*Interaction Effects*				
*ba*_32_	0		0	
*bc*_32_	0		0	
*γ*_32_	-0.07*	(0.04)	0	
Cog. Abil. → Exp.				
*Main Effects*				
*a*_31_	0.3***	(0.09)	0.32***	(0.09)
*c*_31_	0		0	
*β*_31_	0.08^+^	(0.05)	0.09^+^	(0.05)
*Interaction Effects*				
*ba*_31_	-		0	
*bc*_31_	-		0	
*γ*_31_	-		-0.08**	(0.03)
Par. Educ.				
*b*_1_	0.32***	(0.03)	0.32***	(0.03)
*b*_2_	0.2***	(0.02)	0.2***	(0.02)
*b*_3_	0.29***	(0.04)	0.3***	(0.04)
-2LL	14669.95		14664.76	
N	1029		1029	

Standard errors in parentheses. Parameters with ‘0’ without standard error are fixed to 0. Significance: *p* < 0.001:***; *p* < 0.01:**; *p* < 0.05:*; *p* < 0.10:^+^.

Also, the responsiveness of expectations to prior performance is negatively moderated by SES. For students with an average SES level, the responsiveness is *β*_32_ = 0.22 (*p* < 0.001) and a 1 SD increase in SES leads to a reduction of the effect by 0.07 points (*γ*_32_ = −0.07, *p* < 0.05). This interaction is shown in panel A in Fig 2 with high-SES students being less responsive to prior performance than low-SES students resulting in a widening gap among low-performance students as assumed by the compensatory advantage hypothesis (*H*_2_). As in model M3a the SES differences in the responsiveness to cognitive ability are assumed to be zero, the estimated slopes shown in panel B in Fig 2 do not vary by SES.

Model M3b additionally allows the effects of the genetic and shared environmental factors associated with cognitive ability and the responsiveness to cognitive ability to vary by SES. While the negative moderation of the effect of genes unique to expectations remains nearly unchanged (*ba*_22_ = −0.09, *p* < 0.001), the responsiveness to prior performance does not vary anymore by SES. Instead, there is a negative moderation of the responsiveness to cognitive ability by SES. For students with an average SES level, the responsiveness is *β*_31_ = 0.09 (*p* < 0.10) and a 1 SD increase in SES leads to a reduction of the responsiveness of 0.08 points (*γ*_31_ = −0.08, *p* < 0.05). So, the SES differences in the responsiveness to prior performance detected in M3a are completely accounted for by SES differences in the responsiveness to cognitive ability. Panel C in Fig 2 shows that in M3b the responsiveness to prior performance does not depend on SES when accounting for SES differences in the responsiveness to cognitive ability. Panel D in Fig 2 shows that the SES differences in the responsiveness to cognitive ability follow the pattern assumed by the compensatory advantage hypothesis where a lower cognitive ability hardly leads to a downward adjustment of high-SES students’ expectations. So, based on model M3b, both hypotheses *H*_2_ and *H*_3_ need to be rejected as there are no SES differences in the responsiveness of students’ expectations to performance.

## Discussion

As educational expectations are conceptualized as the “strategic center” of educational attainment process [3], studying the formation of expectations contributes to the more general understanding of inequality of opportunity. This study adds to the literature on the formation of educational expectations by asking whether students’ expectations respond to prior educational performance and whether this responsiveness varies by SES. The main contribution of the study is to approach this questions by applying a behavioral genetic design [25] that provides a more accurate estimate of the responsiveness to prior performance by controlling for unobserved genetic and shared environmental confounders. There are two main findings of the study.

First, in line with rational choice arguments [32–35], students’ expectations respond to prior performance. However, there is a substantial degree of genetic and shared environmental confounding. When accounting for these unobserved confounders, the estimated responsiveness is reduced by around 56% from 0.46 to 0.20. Accounting for cognitive ability does not change the estimated responsiveness to performance, meaning that there are two independent ways of updating expectations. However, as students cannot directly observe their cognitive ability, it should be interpreted as a proxy for some unmeasured signal of academic potential. This results supports the notion that signals of academic potential can come from a “variety of sources” [38]. Overall, the multivariate results provide evidence of substantial confounding of the responsiveness to performance by unobserved genetic and shared environmental factors, underlining the relevance of approaches that can control for these types of confounders.

Second, the responsiveness of students’ expectations varies by SES. However, it is not the responsiveness to prior performance that varies by SES. Once SES differences in the responsiveness to cognitive ability are accounted for, the SES differences in the responsiveness to performance disappear. So, while a prior bad performance does lead to a downward adjustment of both, high-SES and low-SES students’ expectations, high-SES students hardly adjust their expectations to lower levels of cognitive ability. This pattern is in line with the compensatory advantage mechanism. So, high-SES students are primarily reluctant to adjust their expectations downward in light of an unobserved negative signal of academic potential proxied by cognitive ability. Interestingly, high-SES students’ expectations are also less responsive to some genetic disposition independent of cognitive ability and performance. Like with cognitive ability, this genetic disposition cannot be observed directly by the students, so it may be seen as a proxy for some genetically influenced signals of academic potential independent of performance and cognitive ability. These could be different personality traits as the genetic correlation with cognitive ability [69] and achievement [70] is only modest meaning that there are substantial genetic influences on personality independent of cognitive ability or achievement. So, while there are no SES differences in the responsiveness to performance, the moderation analysis reveals SES differences in the responsiveness to two unobserved signals of academic potential in line with the compensatory advantage mechanism: one that is proxied by cognitive ability and one that is proxied by the genetic factor unique to educational expectations. As it is beyond the scope of this study, future research could investigate which observable signals of academic potential may mediate these two pathways.

One of this studies’ limitations is that the operationalization of the educational expectations do not take into account tertiary educational outcome, limiting the scope of this study to the formation of expectations for secondary education. Also, while the twin design allowed to control for unobserved genetic and shared environmental confounding of the responsiveness to performance, SES may be not exogenous and may be still affected by unobserved heterogeneity. For example, parents do not only pass on resources but also genes to their offspring. However, other designs such as a nuclear twin family design [71] are needed to estimate this kind of passive gene-environment correlation.

To sum up, this study presents evidence of considerable confounding of the responsiveness of expectations to performance. More than half of the responsiveness to performance is explained by unobserved genetic and shared environmental confounding. But also the SES differences in the responsiveness to performance disappear once SES differences in the responsiveness to cognitive ability are accounted for. So, future research needs to account for these types of confounding to provide a better understanding of the formation of educational expectations and behavioral genetics methods may be a useful tool for this.

## Supporting information

S1 Appendix Appendix. The appendix contains the following information: 1) Coding Scheme of school type; 2) The twin correlations (Table A1); 3) The model comparisons (Tables A2 and A3); 4) Robustness Checks (1.: Accounting for assortative mating (Table A4); 2. Using a composite SES score (Table A5); 3.: No residualization for school type (Table A6); 4.: Controlling for prior expectations (Table A7)).
(PDF)
