# Supplementary material for: Testing for SES differences in the responsiveness of educational expectations in a twin design
Source: PLoS One. 2023 Aug 24;18(8):e0290454. doi: 10.1371/journal.pone.0290454 (PMC10449174; doi:10.1371/journal.pone.0290454)
Supplement: S1 Appendix — The appendix contains the following information: 1) Coding Scheme of school type; 2) The twin correlations (Table A1); 3) The model comparisons (Tables A2 and A3); 4) Robustness Checks (1.: Accounting for assortative mating (Table A4); 2. Using a composite SES score (Table A5); 3.: No residualization for school type (Table A6); 4.: Controlling for prior expectations (Table A7)). (PDF) [file pone.0290454.s001.pdf]

Appendix

Coding Scheme of school type variable

For the analysis, I residualized the school grades for the visited school type which is coded as follows: 1) Primary School ("Grundschule"); 2) Lower secondary school ("Hauptschule"); 3) Intermediate secondary school ("Realschule"); 4) Integrated secondary school, i.e. a school track combining lower and intermediate secondary school; 5) Comprehensive school ("Gesamtschule"); 6) Upper secondary school ("Gymnasium"); 7) Other. As the robustness check presented in A6 in the appendix shows, results do not change without residualization of school grades.

Twin Correlations

	**MZ**	**DZ**
**Expectations**	0.73***	0.41***
**Avg. Grades**	0.75***	0.49***
**Cog. Abil.**	0.64***	0.43***

Table A1: Twin correlations. Significance: $p < 0.001$:***; $p < 0.01$:**; $p < 0.05$:*.

Model comparisons

**Baseline**	**Comparison**	**No. Par.**	**-2LL**	**df**	**Δ-2LL**	**p**
M2a Full	-	15	15311.08	6116	-	-
M2a Full	**M2a Reduced 1**	**14**	**15311.13**	**6117**	**0.05**	**0.83**
M2b Full	-	17	15292.67	6114	-	-
M2b Full	**M2b Reduced 1**	**16**	**15292.67**	**6115**	**0**	**1**
M2c Full	-	23	14924.8	6108	-	-
M2c Full	**M2c Reduced 1**	**21**	**14924.93**	**6110**	**0.13**	**0.94**
M2d Full	-	26	14682.67	6105	-	-
M2d Full	**M2d Reduced 1**	**21**	**14684.51**	**6110**	**1.83**	**0.87**
M3a Full	-	32	14665.75	6099	-	-
M3a Full	M3a Reduced 1	25	14666.98	6106	1.23	0.99
M3a Reduced 1	**M3a Reduced 2**	**23**	**14669.95**	**6108**	**2.98**	**0.23**
M3b Full	-	35	14660.05	6096	-	-
M3b Full	M3b Reduced 1	28	14661.35	6103	1.29	0.99
M3b Reduced 1	**M3b Reduced 2**	**23**	**14664.76**	**6108**	**3.41**	**0.64**

Table A2: Model comparisons. For univariate models M1a, M1b and M1c, the full models are the best fitting models.

**Model**	**Specification**
M2a Reduced 1	Drop c_{33}
M2b Reduced 1	Drop c_{33}
M2c Reduced 1	Drop c_{33}, a_{32}
M2d Reduced 1	Drop c_{33}, a_{32}, c_{31} c_{21}, c_{32}
M3a Reduced 1	Drop bc_{33}, c_{33}, ba_{32}, a_{32}, c_{21}, c_{31}, bc_{32}
M3a Reduced 2	Drop c_{32}, be_{33}
M3b Reduced 1	Drop bc_{33}, c_{33}, ba_{32}, a_{32}, c_{21}, bc_{31}, ba_{31}
M3b Reduced 2	Drop c_{31}, bc_{32}, c_{32}, be_{33}, γ_{32}

Table A3: Model specifications.

Robustness checks

Assortative mating

Assortative mating increases the genetic correlation of DZ twins (rG_{DZ}). Here, the results of robustness checks are shown for different degrees of assortative mating ($rG_{DZ} = 0.60$ and $rG_{DZ} = 0.65$). The robustness check supports the main results.

	**M3bR1a**		**M3bR1b**	
Exp.				
Main Effects				
a_{33}	0.67***	(0.03)	0.67***	(0.03)
c_{33}	0		0	
e_{33}	0.52***	(0.02)	0.53***	(0.02)
Interaction Effects				
ba_{33}	-0.09***	(0.03)	-0.09***	(0.03)
bc_{33}	0		0	
be_{33}	0		0	
Avg. Grades → Exp.				
Main Effects				
a_{32}	0		0	
c_{32}	0		0	
β_{32}	0.23***	(0.05)	0.25***	(0.04)
Interaction Effects				
ba_{32}	0		0	
bc_{32}	0		0	
γ_{32}	0		0	
IQ → Exp.				
Main Effects				
a_{31}	0.28**	(0.09)	0.24***	(0.07)
c_{31}	0		0	
β_{31}	0.09*	(0.05)	0.11*	(0.05)
Interaction Effects				
ba_{31}	0		0	
bc_{31}	0		0	
γ_{31}	-0.08**	(0.03)	-0.08**	(0.03)
Par. Educ.				
b_1	0.32***	(0.03)	0.32***	(0.03)
b_2	0.2***	(0.02)	0.2***	(0.02)
b_3	0.3***	(0.04)	0.29***	(0.03)
-2LL	14670.72		14676.2	
N	1029		1029	

Table A4: Results of robustness check 1. M3bR1 assumes a genetic correlation for DZ twins of 0.60 and M3bR2 of 0.65. Standard errors in parentheses. Parameters with '0' without standard error are fixed to 0. Significance: $p < 0.001$:***; $p < 0.01$:**; $p < 0.05$:*; $p < 0.10$:$^{+}$.

Composite SES score

Shown are results when using a composite score instead of parental years of education to measure parental SES. The composite score is based on the three central dimensions of SES: parental education, occupational status (ISEI) [1] and income. It is calculated as the factor score in a confirmatory factor analysis. The robustness check supports the main results.

	**M3bR2**	
Exp.		
Main Effects		
a_{33}	0.66***	(0.04)
c_{33}	0	
e_{33}	0.5***	(0.02)
Interaction Effects		
ba_{33}	-0.11***	(0.03)
bc_{33}	0	
be_{33}	0	
Avg. Grades → Exp.		
Main Effects		
a_{32}	0	
c_{32}	0	
β_{32}	0.19***	(0.06)
Interaction Effects		
ba_{32}	0	
bc_{32}	0	
γ_{32}	0	
IQ → Exp.		
Main Effects		
a_{31}	0.34***	(0.09)
c_{31}	0	
β_{31}	0.08^{+}	(0.05)
Interaction Effects		
ba_{31}	0	
bc_{31}	0	
γ_{31}	-0.06*	(0.03)
Par. Educ.		
b_1	0.35***	(0.03)
b_2	0.22***	(0.02)
b_3	0.31***	(0.04)
-2LL	14662.57	
N	1039	

Table A5: Results of robustness check 2 using a composite score to measure parental SES. Standard errors in parentheses. Parameters with '0' without standard error are fixed to 0. Significance: $p < 0.001$:***; $p < 0.01$:**; $p < 0.05$:*; $p < 0.10$:$^{+}$.

Without residualization for school type

	M3bR3	
Exp.		
Main Effects		
a_{33}	0.65***	(0.04)
c_{33}	0	
e_{33}	0.5***	(0.02)
Interaction Effects		
ba_{33}	-0.09***	(0.03)
bc_{33}	0	
be_{33}	0	
Avg. Grades → Exp.		
Main Effects		
a_{32}	0	
c_{32}	0	
β_{32}	0.19**	(0.06)
Interaction Effects		
ba_{32}	0	
bc_{32}	0	
γ_{32}	0	
IQ → Exp.		
Main Effects		
a_{31}	0.34***	(0.09)
c_{31}	0	
β_{31}	0.08^{+}	(0.05)
Interaction Effects		
ba_{31}	0	
bc_{31}	0	
γ_{31}	-0.08**	(0.03)
Par. Educ.		
b_1	0.32***	(0.03)
b_2	0.22***	(0.02)
b_3	0.31***	(0.04)
-2LL	14804.05	
N	1029	

Table A6: Results of robustness check 3 without residualization for school type. Standard errors in parentheses. Parameters with '0' without standard error are fixed to 0. Significance: $p < 0.001$:***; $p < 0.01$:**; $p < 0.05$:*; $p < 0.10$:$^{+}$.

Controlling for prior expectations

	M3bR4	
Exp.		
Main Effects		
a_{33}	0.6***	(0.03)
c_{33}	0	
e_{33}	0.52***	(0.02)
Interaction Effects		
ba_{33}	-0.12***	(0.03)
bc_{33}	0	
be_{33}	0	
Avg. Grades → Exp.		
Main Effects		
a_{32}	0	
c_{32}	0	
β_{32}	0.15***	(0.04)
Interaction Effects		
ba_{32}	0	
bc_{32}	0	
γ_{32}	0	
IQ → Exp.		
Main Effects		
a_{31}	0.19*	(0.08)
c_{31}	0	
β_{31}	0.11*	(0.05)
Interaction Effects		
ba_{31}	0	
bc_{31}	0	
γ_{31}	-0.05^{+}	(0.03)
Par. Educ.		
b_1	0.3***	(0.03)
b_2	0.16***	(0.02)
b_3	0.2***	(0.04)
-2LL	18768.21	
N	1029	

Table A7: Results of robustness check 4 controlling for prior expectations. Standard errors in parentheses. Parameters with '0' without standard error are fixed to 0. Significance: $p < 0.001$:***; $p < 0.01$:**; $p < 0.05$:*; $p < 0.10$:$^{+}$.

References

[1] Ganzeboom HBG, De Graaf PM, Treiman DJ. A standard international socio-economic index of occupational status. Social Science Research. 1992;21(1):1–56. doi:10.1016/0049-089X(92)90017-B.
